# Aortic Calcification Can Serve as an Independent Predictor of Arteriovenous Fistula Failure in Maintenance Hemodialysis Patients

**DOI:** 10.7759/cureus.95213

**Published:** 2025-10-23

**Authors:** Zhuang Feng, Wang Yingdeng, Xian Shuli

**Affiliations:** 1 Department of Nephrology, Ninth People’s Hospital, School of Medicine, Shanghai Jiao Tong University, Shanghai, CHN

**Keywords:** aortic calcification, aortic calcification score, arteriovenous fistula, hemodialysis, primary patency rate

## Abstract

Background

This study aimed to observe the relationship between aortic calcification and the primary patency rate of new autologous internal fistulas in maintenance hemodialysis patients and to explore the possibility of predicting the dysfunction of autologous internal fistulas using the aortic calcification score.

Methodology

A total of 75 patients who underwent autologous arteriovenous fistula and hemodialysis for the first time in our hospital between January 2016 and December 2019 were selected. Abdominal aortic calcification index (ACI) was recorded at the time of internal fistula establishment. Patients were divided into high- and low-calcification groups based on the ACI results. The primary patency rate of autologous internal fistulas in the two groups was observed over five years.

Results

Of the 75 patients, 70 (93.33%) had varying degrees of aortic calcification, with 37 patients in the high calcification group (ACI > 10%). During the five-year follow-up period, 64 patients developed internal fistula dysfunction due to various causes, including 35 cases of thrombosis and 19 cases of fistula stenosis. At 12, 36, and 60 months, the primary patency rates of internal fistulas in the low calcification group were 92.5%, 57.5%, and 17.5%, respectively, whereas those in the high calcification group were 85.7%, 30.4%, and 9.1%, respectively. The difference in primary patency rates between the two groups was statistically significant after five years (F = 4.443, p = 0.035). Cox analysis showed that autologous internal fistula dysfunction was related to ACI (hazard ratio = 2.114, 95% confidence interval = 1.146-3.899, p = 0.017).

Conclusions

A higher aortic calcification score was associated with dysfunction of the autologous arteriovenous fistula within five years. Patients with higher aortic calcification scores require more frequent monitoring of the internal fistula function and early intervention to prolong the service life of the internal fistula.

## Introduction

Autologous arteriovenous fistulas (AVFs) have been recommended as the preferred vascular access for hemodialysis patients in clinical practice guidelines, offering advantages such as prolonged patient survival and reduced dialysis complications. However, AVFs have issues such as long maturation times and high failure rates. Vascular access dysfunction causes high mortality and hospitalization rates in patients [1]. Notably, vascular calcification, including calcification of the vascular access and aortic arch, is common in dialysis patients [2], and pre-existing vascular access artery calcification is associated with poorer AVF outcomes in these populations [3]. Therefore, the question arises as to whether pre-existing large artery calcification also has predictive value for AVF dysfunction. However, relevant studies are scarce. This study primarily observed the predictive factors for first AVF failure within five years, particularly the impact of the abdominal aortic calcification index (ACI) on AVF patency after the initial establishment of AVF in hemodialysis patients.

## Materials and methods

Study participants

From January 2016 to December 2019, patients with stage 5 chronic kidney disease (CKD) who underwent initial AVF surgery and hemodialysis at the Ninth People’s Hospital were included in this retrospective observational study. A total of 88 patients were initially enrolled, excluding those with a follow-up duration <3 months after dialysis initiation (n = 5), immature fistulas (n = 3), transfer from peritoneal dialysis (n = 3), and those without chest or abdominal CT scan records (n = 2), leaving 75 patients for the final analysis. The patients’ ages ranged from 22 to 86 years, with 26 males and 49 females. The maturation and use of AVF in the included patients were observed over five years post-surgery. This study was approved by the Ethics Committee of Ninth People’s Hospital, School of Medicine, Shanghai Jiao Tong University (approval number: SH9H-2022-T95-1).

Clinical data

We collected clinical data from the patients during the establishment of AVF, including age, sex, underlying diseases (diabetes, hypertension, and severe cardiovascular or cerebrovascular diseases), systolic blood pressure, laboratory test data, and aortic calcification score. A history of typical angina pectoris, myocardial infarction, coronary artery bypass surgery or intervention, or cerebrovascular events, such as cerebral hemorrhage or infarction, was considered a history of severe cardiovascular or cerebrovascular diseases. All laboratory measurements, including hemoglobin, albumin, alkaline phosphatase, serum calcium, phosphorus, parathyroid hormone, and low-density lipoprotein, were performed using standardized and automated methods in our hospital’s laboratory.

Assessment of the aortic calcification index

Non-contrast CT scans of the chest or abdomen (with a standardized slice thickness of 5 mm) were obtained within a perioperative period of one month relative to AVF creation. Ten consecutive aortic planes were examined at 1 cm intervals. Each plane was radially subdivided into 12 sectors. The number of sectors with calcified areas showing a density ≥100 HU was calculated. The sum was divided by 120 and multiplied by 100 to express it as a percentage [4]. To unify the standards, we selected the area 10 cm below the outlet of the descending aorta as the baseline for assessing calcification. Based on the percentage obtained, patients with ≤10% calcification were classified into the low calcification group, and those with >10% calcification were classified into the high calcification group.

Clinical outcome determination

All AVFs were established by three experienced doctors (defined as A + B or A + C). The strategy for AVF creation was based on physical examination, ultrasound diagnosis, and physician experience, starting from the distal end of the nondominant arm and proceeding to the proximal end and nondominant arm as per the guidelines. Fistula life was defined as the time from the first puncture to the first fistula occlusion or the need for any interventional or surgical intervention [5].

Statistical analysis

Data were statistically analyzed using SPSS Statistics 28.0.1.0 software (IBM Corp., Armonk, NY, USA). Measurement data are expressed as mean ± standard deviation, and comparisons between groups were performed using the t-test. Count data are expressed as numbers and percentages, and comparisons between groups were performed using the chi-square test. The Cox proportional hazards model was used to identify factors associated with decreased access patency, and the Kaplan-Meier method was used to determine the primary patency rate curve of the AVF. Statistical significance was set at p-values <0.05.

## Results

Baseline characteristics

A total of 75 long-term maintenance hemodialysis patients were included in this study, with 49 males and 26 females, with an average age of 64.0 ± 14.9 years. AVFs were established in the elbow in 19 (25.33%) patients and in the forearm in the remaining 56 (74.67%) patients. Five patients showed no calcification on CT scans, whereas the remaining 70 (93.33%) patients had varying degrees of aortic calcification. Based on the ACI at the time of fistula establishment, the patients were divided into a low calcification group (≤10%) and a high calcification group (>10%), with 21 patients with ACI > 20, 16 patients with 10 < ACI ≤ 20, and 16 patients with 5 < ACI ≤ 10. The baseline data for the two groups are presented in Table 1 and Table 2. Patients in the high calcification group were generally older and had more comorbidities, such as cardiovascular and cerebrovascular diseases and diabetes, than those in the low calcification group. However, there were no statistically significant differences in common laboratory indicators (such as hemoglobin, calcium, phosphorus, parathyroid hormone, and low-density lipoprotein) between the two groups (Table 2).

**Table 1:** General Information of patients grouped by ACI. ACI: abdominal aortic calcification index

Characteristics	Total (n = 75)	ACI		F	P-value
		Low calcification group	High calcification group		
Gender, n (%)	75	40	35		
Male	49 (65.3)	26 (53.1)	23 (46.9)	0.267	0.952
Female	26 (34.7)	14 (53.8)	12 (46.2)		
Basic diseases					
Hypertension, n (%)	68 (90.7)	35	33	0.346	0.314
Diabetes, n (%)	36 (48.0)	14	22	6.123	0.016
Cardiovascular and cerebrovascular diseases, n (%)	28 (37.3)	8	18	8.889	0.004
Fistula location, n (%)					
Forearm	56 (74.67)	31	25	0.366	0.542
Mid-upper arm	19 (25.33)	9	10		

**Table 2:** Baseline characteristics of patients grouped by ACI. Data are presented as mean ± SD or n (%). ACI: abdominal aortic calcification index; SBP: systolic blood pressure; iPTH: intact parathyroid hormone; LDL: low-density lipoprotein; HDL: high-density lipoprotein

Characteristics	Total	ACI		t-value	P-value
		Low calcification group	High calcification group		
Age (years)	64.0 ± 14.9	55.4 ± 14.0	73.9 ± 8.3	7.225	0.009
SBP (mmHg)	154 ± 35	148 ± 31	157 ± 36	0.542	0.467
ACI (%)	15.2 ± 15.9	4.5 ± 3.0	27.4 ± 15.8	40.498	0.001
Laboratory examination					
Hemoglobin (g/L)	82.0 ± 18.5	80.2 ± 19.3	83.0 ± 18.5	0.066	0.802
Albumin (g/L)	33.9 ± 6.2	34.4 ± 6.4	33.7 ± 6.2	0.482	0.88
Serum uric acid (mmol/L)	456.6 ± 130.6	504.1 ± 126.7	418.2 ± 132.0	0.465	0.503
Serum creatinine (mmol/L)	761.4 ± 223.5	813.9 ± 319.4	721.4 ± 223.5	4.523	0.084
Calcium (mmol/L)	1.98 ± 0.27	2.0 ± 0.27	1.96 ± 0.55	0.046	0.087
Phosphate (mmol/L)	1.92 ± 0.60	1.98 ± 0.62	1.72 ± 0.79	3.596	0.062
Magnesium (mmol/L)	0.90 ± 0.16	0.91 ± 0.17	0.81 ± 0.33	5.431	0.050
iPTH (pg/mL)	220.38 ± 144.3	237.4 ± 145.1	200.8 ± 142.9	0.015	0.902
LDL (mmol/L)	2.61 ± 1.09	2.80 ± 1.18	2.27 ± 0.95	0.381	0.537
HDL (mmol/L)	0.91 ± 0.39	0.95 ± 0.51	0.79 ± 0.27	3.682	0.052
Lipoprotein a (mmol/L)	0.29 ± 0.26	0.31 ± 0.25	0.28 ± 0.29	2.195	0.141

Manifestations of autologous arteriovenous fistula dysfunction

Table 3 presents the detailed characteristics of autologous AVFs that required intervention. During the five-year follow-up, 64 patients developed AVF dysfunction due to various causes, including thrombotic fistula occlusion in 35 patients, inadequate blood flow due to local stenosis in 19 patients, intervention due to limited puncture sites in four patients, venous aneurysm resection in two patients, increased venous pressure in two patients, fistula infection in one patient, and steal syndrome in one patient. All patients underwent interventional or open surgical treatment after hospitalization.

**Table 3:** Demographic and clinical characteristics of patients with arteriovenous fistula dysfunction (n = 64)‌.

Variable	Value (N = 64)
Age (years)	64.54 ± 14.72
Gender, n (%)	
Male	39 (60.94%)
Female	25 (39.06%)
Fistula location, n (%)	
Forearm	49 (76.56%)
Mid-upper arm	15 (23.44%)
Manifestations of dysfunction, n (%)	
Thrombosis	35 (54.69%)
Stenosis	19 (29.69%)
Limited puncture sites	4 (6.25%)
Venous hypertension	2 (3.13%)
Aneurysmal dilation	2 (3.13%)
Steal syndrome	1 (1.56%)
Infection	1 (1.56%)
Interventions, n (%)	
Open surgery	45 (70.31%)
Balloon angioplasty	14 (21.88%)
Semi-permanent catheter	5 (7.81%)

Factors related to autologous arteriovenous fistula dysfunction

Multivariate Cox analysis revealed that the ACI was associated with an increased risk of AVF failure (95% confidence interval (CI) = 1.146-3.899; p = 0.017). Factors such as diabetes, cardiovascular and cerebrovascular diseases, serum albumin, low-density lipoprotein, serum phosphorus, and parathyroid hormone levels were not significantly associated with the primary patency rate (p > 0.05) (Table 4).

**Table 4:** Multivariable Cox analysis of factors associated with loss of primary patency in AVF (n = 75). The only statistically significant predictor was ACI > 10% (HR = 2.114, 95% CI = 1.146-3.899, p = 0.017), suggesting arterial calcification is an independent risk factor for AVF primary patency loss. All other variables showed no significant association in this multivariable model. AVF: arteriovenous fistulas; ALB: serum albumin; Hb: hemoglobin; P: serum phosphorus; iPTH: intact parathyroid hormone; LDL: low-density lipoprotein; ACI: abdominal aortic calcification index; SE = standard error; HR = hazard ratio; CI = confidence interval

Variable	B	SE	Wald	P-value	HR	95% CI for HR	
						Lower	Upper
ALB <35 g/L	-0.144	0.380	0.144	0.705	1.155	0.548	2.435
Hb <90 g/L	-0.189	0.455	0.173	0.677	0.827	0.339	2.018
Age >65 years	0.044	0.268	0.026	0.871	0.827	0.617	1.768
P >1.78 mmol/L	0.042	0.319	0.018	0.894	1.043	0.559	1.948
iPTH >300 µmol/L	-0.173	0.339	0.260	0.610	0.842	0.433	1.634
LDL >3.12 mmol/L	0.308	0.325	0.897	0.344	1.360	0.720	2.570
Diabetes (yes)	-0.201	0.352	0.326	0.568	0.818	0.410	1.631
Cardiovascular or cerebrovascular diseases (yes)	-0.360	0.339	1.123	0.289	0.698	0.359	1.357
ACI >10%	0.749	0.312	5.746	0.017	2.114	1.146	3.899

**Figure 1:**
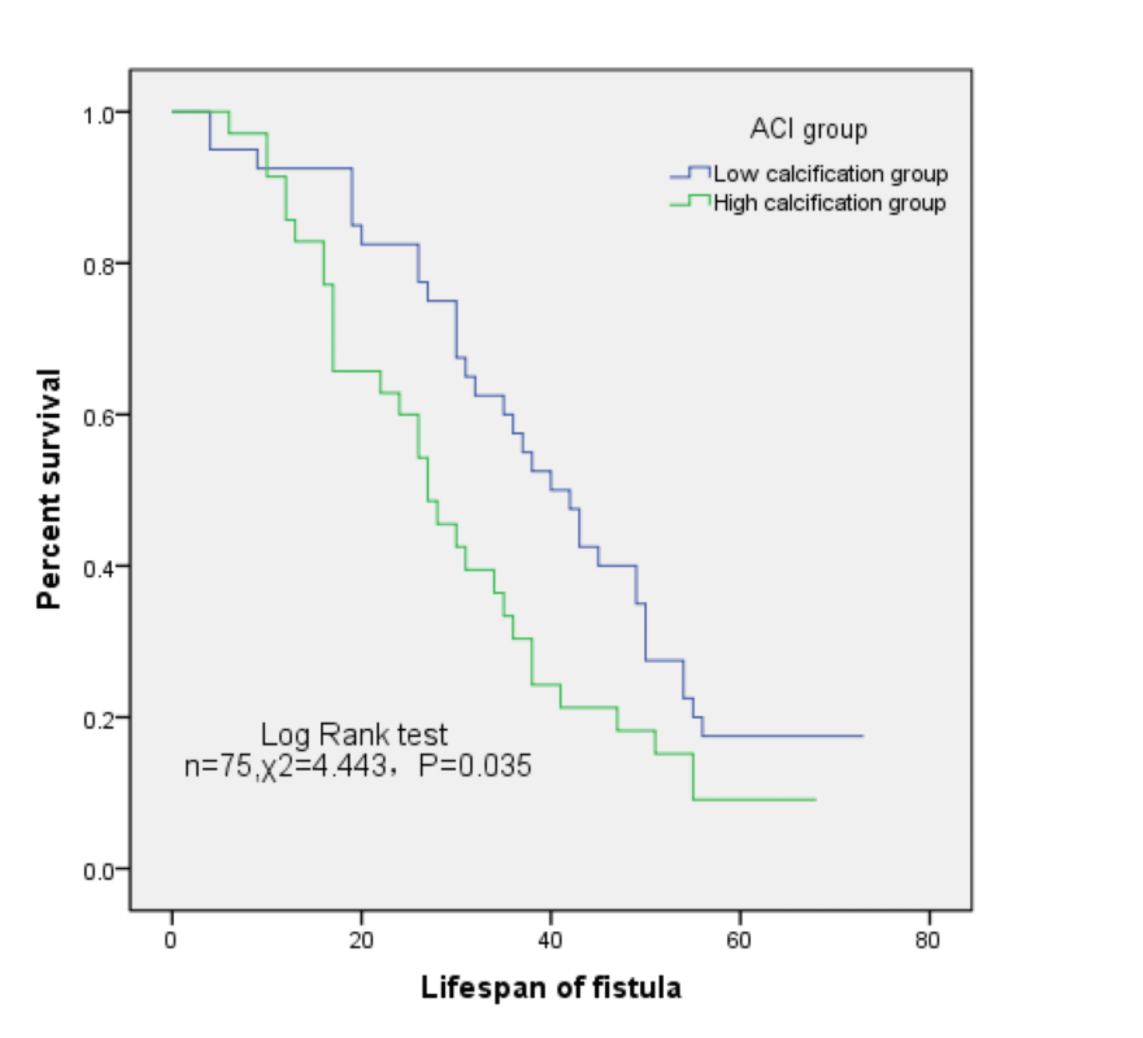
Kaplan-Meier survival curves of autologous arteriovenous fistula in different calcification groups. ACI: abdominal aortic calcification index

At 12, 36, and 60 months, the primary patency rates of internal fistulas in the low calcification group were 92.5%, 57.5%, and 17.5%, respectively, whereas those in the high calcification group were 85.7%, 30.4%, and 9.1%, respectively. Kaplan-Meier analysis confirmed that patients with higher ACI had significantly lower survival rates of AVF than those with lower ACI (log-rank test, F = 4.443, p = 0.035) (Figure 1), indicating a higher risk of new fistula dysfunction (hazard ratio = 1.661, 95% CI = 0.997-2.165; p = 0.035).

## Discussion

Vascular calcification is commonly observed among patients with CKD. In a study of 280 non-dialysis CKD patients who underwent X-ray examinations, 72% were found to have varying degrees of abdominal aortic calcification [6]. In our study, only five (6.7%) patients showed no calcification, which may be related to the fact that all our patients had stage 5 CKD and thus had a higher calcification rate. Moreover, CT scans are more sensitive in detecting calcified lesions. Many studies have explored factors affecting the long-term patency of AVF [7], including demographic factors, duration of diabetes, diameter of the target vessel, and any signs of atherosclerosis. Calcified vessels directly affect the maturation and use of an AVF. Choi et al. obtained arterial specimens from 114 patients undergoing AVF surgery and followed them up for one year, finding that patients with arterial microcalcification had significantly lower AVF patency rates than those without calcification [8]. The specific mechanism may involve increased vascular damage and neointimal hyperplasia owing to enhanced shear stress caused by vascular calcification [9]. Additionally, factors related to vascular calcification, such as low fetuin-A, high osteopontin, and bone morphogenetic protein 7, are associated with AVF complications, but not with arteriovenous graft complications, suggesting that calcification plays a role in the pathogenesis of AVF failure [10].

Pre-existing vascular calcification before AVF establishment leads to adverse outcomes for the fistula, although opinions differ [11]. Moreover, vascular resources are valuable during surgery, and it is not feasible to easily obtain specimens for pathological examination. Pure X-ray imaging has poor sensitivity for mild vascular calcification and is rarely used. Previous studies have shown that CT technology is more sensitive than plain radiography in detecting peripheral and aortic vascular calcification and that the results of CT scans assessing abdominal aortic vascular calcification have the highest predictive value for cardiovascular events and mortality in patients [12]. Our study used chest and abdominal CT scans and found that aortic calcification is prevalent in patients with stage 5 CKD, and the proportion of patients with first AVF failure within five years was significantly lower in those with an ACI ≤10% than in those with an ACI >10%. The degree of aortic calcification is associated with adverse AVF outcomes.

Advanced age, diabetes, and cardiovascular and cerebrovascular diseases are high-risk factors for aortic calcification; the more high-risk factors are present, the more severe the aortic calcification. These high-risk factors are also important causes of AVF dysfunction [13]; however, this study did not show any correlation between these independent factors and AVF failure. Furthermore, aortic calcification may be a marker of systemic vascular calcification and atherosclerosis, often correlated with the degree of coronary artery calcification, heart valve calcification, intracranial artery calcification [14], and microcalcification of vascular access [15]. As previously reported [3,8], increased radial artery intima-media thickness and pre-existing vascular access calcification may predict AVF dysfunction in patients with end-stage renal disease. In our study, the three-year primary patency rate of fistulas in the high calcification group was only 45.9%, which was significantly lower than that in the low calcification group, indicating that severe aortic calcification may indirectly suggest an increased risk of AVF failure. ACI can serve as a noninvasive method for assessing the outcomes of newly established AVF in dialysis patients.

Impaired vascular dilation and outward remodeling due to arteriosclerosis are suspected causes of AVF maturation failure and restenosis. Pre-existing calcification may lead to arteriosclerosis and hinder the outward remodeling of inflow arteries, thereby limiting their dilation and sufficient blood flow, eventually leading to fistula dysfunction [16]. Additionally, mechanical stretching due to intraluminal pressure acts on endothelial and smooth muscle cells after vascular calcification, leading to intimal hyperplasia [17]. Furthermore, blood flow disturbances in AVF may be enhanced in calcified AVF, promoting dysfunctional endothelial cell phenotypes, inducing the secretion of pro-inflammatory mediators such as nuclear factor-κB, and increasing the expression of genes related to oxidation and proliferation [18]. Studies have also shown that thrombin plays a role in the pathophysiology of vascular calcification [19] and promotes thrombosis in arteriovenous fistulas.

Hypomagnesemia is a well-established risk factor for vascular calcification and endothelial dysfunction [20], both of which represent core pathological mechanisms underlying AVF failure. Our study also indicated that serum magnesium levels approached borderline significance in relation to AVF failure, suggesting that hypomagnesemia may contribute to the impairment of AVF. However, the strength and consistency of this association remain somewhat uncertain. Therefore, hypomagnesemia may represent a potential and modifiable risk factor, warranting further larger-scale studies to clarify the specific role of magnesium levels and their clinical significance. Elevated parathyroid hormone levels increase the risk of atherosclerotic disease [21] and are considered a risk factor for AVF patency loss [22]. However, this association was not observed in this study. The parathyroid hormone levels in the high calcium group were not significantly elevated, and Cox analysis also did not show a significant correlation between fistula failure and high parathyroid hormone levels, similar to a study from Taiwan [23]. This may be because our patients had stage 5 CKD and had not yet started dialysis. Most patients had mild-to-moderate increases in parathyroid hormone levels for a relatively short duration, which had not yet significantly affected the systemic bone mineral metabolism. Additionally, some studies suggest that higher calcium and lower intact parathyroid hormone levels may be associated with higher aortic calcification grades owing to reduced bone calcium buffering capacity [24]. Low albumin [25] and high phosphorus [26] levels are also considered risk factors for autologous fistula failure, but we did not find significant correlations in our study.

Our study had several limitations. First, we selected patients who had undergone fistula surgery and had been on stable dialysis for >3 months, excluding those who chose other vascular accesses, were lost to follow-up after surgery, or had shorter survival times. Therefore, the short-term lifespan of the fistulas in the included patients was higher and may not represent the overall dialysis population, potentially introducing a bias. Second, this was a single-center, retrospective study, and some factors were not recorded and analyzed in detail, including vessel diameter, fistula puncture time, or inflammatory markers. These factors may also be associated with autologous fistula dysfunctions. The results of this study support ACI as a promising predictor of fistula failure, but further confirmation is needed through the design of more rigorous and larger prospective studies.

## Conclusions

Aortic calcification is prevalent in patients with end-stage renal disease, and a higher ACI is associated with AVF dysfunction within five years. Stratification of the ACI through CT scans can allow clinicians to monitor and intervene more closely in fistulas with a high risk of failure, thereby prolonging their service life.

## Appendices

**Table 5:** STROBE statement.

Section/Topic	Item number	Recommendation	Reported on page number
Title and abstract	1	(a) Indicate the study’s design with a commonly used term in the title or the abstract	1
		(b) Provide in the abstract an informative and balanced summary of what was done and what was found	1
Introduction			
Background/Rationale	2	Explain the scientific background and rationale for the investigation being reported	2
Objectives	3	State specific objectives, including any prespecified hypotheses	2
Methods			
Study design	4	Present key elements of the study design early in the paper	2
Setting	5	Describe the setting, locations, and relevant dates, including periods of recruitment, exposure, follow-up, and data collection	2
Participants	6	(a) Cohort study—Give the eligibility criteria, and the sources and methods of selection of participants. Describe methods of follow-up. Case-control study—Give the eligibility criteria, and the sources and methods of case ascertainment and control selection. Give the rationale for the choice of cases and controls. Cross-sectional study—Give the eligibility criteria, and the sources and methods of selection of participants	2
		(b) Cohort study—For matched studies, give matching criteria and number of exposed and unexposed. Case-control study—For matched studies, give matching criteria and the number of controls per case	2
Variables	7	Clearly define all outcomes, exposures, predictors, potential confounders, and effect modifiers. Give diagnostic criteria, if applicable	2, 3
Data sources/Measurement	8*	For each variable of interest, give sources of data and details of methods of assessment (measurement). Describe comparability of assessment methods if there is more than one group	2, 3
Bias	9	Describe any efforts to address potential sources of bias	3
Study size	10	Explain how the study size was arrived at	3
Quantitative variables	11	Explain how quantitative variables were handled in the analyses. If applicable, describe which groupings were chosen and why	3
Statistical methods	12	(a) Describe all statistical methods, including those used to control for confounding	3
		(b) Describe any methods used to examine subgroups and interactions	3
		(c) Explain how missing data were addressed	3
		(d) Cohort study—If applicable, explain how loss to follow-up was addressed. Case-control study—If applicable, explain how the matching of cases and controls was addressed. Cross-sectional study—If applicable, describe analytical methods taking account of sampling strategy	3
		(e) Describe any sensitivity analyses	
